# Phase 0 clinical trials: towards a more complete ethics critique

**DOI:** 10.3332/ecancer.2012.248

**Published:** 2012-03-27

**Authors:** T Patrick Hill

**Affiliations:** Senior Policy Fellow, The Edward J. Bloustein School of Planning and Public Policy, Rutgers, The State University of New Jersey, New Jersey, USA

## Abstract

In efforts to modernise the entire drug-development process, making it more efficient, less costly, and ultimately of real benefit to patients, The Federal Drug Administration (FDA) authorised the use of exploratory IND or early Phase I (Phase 0) studies. Quite different in structure from Phase I, II, and III studies, the Phase 0 construct understandably poses a set of ethical problems not seen in the other research phases and so far not adequately addressed by ethicists. In an effort to deal with this deficiency, this paper proposes an ethics critique, based not on the usual concept of benefit, but on the means–end relation, and placed within an ethic of science derived from the practice of science.

To date, the ethics analysis of Phase 0 clinical trials has remained incomplete [1–3]. Focusing, for the most part, on the Phase 0 construct itself, the analysis has neglected the larger economic context in relation to which Phase 0 trials must be understood for any complete analysis.

## The Pipeline Problem

One of the clearest delineations of this context is found in the *Critical Path Initiative* issued by the Federal Drug Administration (FDA) in March 2004 [4] in its effort to modernise the entire drug-development process. Prompting this effort was what the FDA called the pipeline problem or the slowdown in innovative medical therapies available to patients. Despite the revolution in biomedical science that had raised expectations for prevention, treatment and cure of serious illness, the FDA acknowledged a growing concern that many of the recent basic science discoveries might not translate rapidly into more effective, safe and affordable medical interventions for patients. “This is because the current medical product development path is becoming increasingly challenging, inefficient and costly” [5]. As evidence of this, the FDA pointed to a serious decline in the number of new drug and biologic applications then being submitted to the agency, while the number of innovative medical-device applications had also become fewer. At the same time, research and development costs were increasing alarmingly. As the FDA observed, in the face of escalating costs, innovators are inclined to focus on developments expected to have high financial rewards. As a consequence, “[d]eveloping products targeted for important public health needs (e.g., counterterrorism), less common diseases, prevalent third world diseases, prevention indications, or individualised therapy is becoming increasingly challenging” [6]. The root of the problem, according to the FDA, is found in the fact that the applied sciences that are required for the development of medical products have not kept pace with the extraordinary levels of progress achieved by basic research. “Not enough applied scientific work has been done to create new tools to get fundamentally better answers about how the safety and effectiveness of new products can be demonstrated in faster time frames, with more certainty, and at lower costs” [7]. In other words, according to the FDA, investigators find themselves having to use outdated research concepts and methods, resulting in unsustainable levels of failure of medical products entering clinical studies. This failure rate in turn adds to the cost of research so that developers confront a situation of diminishing returns on investment. Profits from a dwindling number of successful products are in effect subsidising an accelerating number of failing initiatives. And even when the product proves to be successful, it does so only as the culmination of a long, expensive and inefficient process, notable for its cumbersome methods of assessment.

In its discussion of negotiating the critical path, the FDA points out that even after 10 years of pre-clinical screening and evaluation, a new product had at that time only an 8 per cent chance of reaching the market. This probability compares unfavourably with the historical rate of success of around 14 per cent. By way of illustration, the FDA noted that a drug entering a Phase I study in 2000 was not more likely to reach the market than one entering a Phase I study in 1985. That the inability to identify early new drugs likely to fail is costly can be seen in the fact that improving our ability to predict failures by 10 per cent before going to a clinical trial would save US$100 million in development costs per drug.

However, given the scientific and technical dimensions of the critical path, making this degree of improvement is not easy. As the FDA acknowledged, whether they are considering drugs, devices, or biologicals, medical-product developers have to navigate three indispensable phases that constitute the critical path as it leads from a scientific innovation to a marketable product. They are assessment of safety at both a pre-clinical and clinical level; confirmation of medical usefulness, again at both a pre-clinical and clinical level; and confirmation of industrialisation or the translation of a prototype into a product capable of being mass produced. As designed historically, the critical path assumed, according to the FDA, an interdependence among all three dimensions, even though there was no assurance of success in any of them.

To address what can only be described as a systemic problem, the FDA insisted that “[a] new product-development toolkit – containing powerful new scientific methods, such as animal or computer-based predictive models, biomarkers for safety and effectiveness, and new clinical evaluation techniques – is urgently needed to improve predictability and efficiency along the critical path from laboratory concept to commercial product” [8].

In concluding its critical path report, The FDA argued that progress along the path requires more than better medical ideas. It must also include producing reliable, safe and effective treatments for patients at affordable prices. “This is an essential step in achieving more timely, affordable, and predictable access to therapies based on the latest biomedical insights – that so far are having little impact on patient care [9]. To this end, the FDA declared that since it is involved in setting standards for the development of new medical products, it must, as part of that involvement, take a proactive role in the application of the best science to inform the development process and to require that development standards, in keeping with “best science” practice, are so rigorous and efficient as to result in the greatest benefit possible for the public’s health.

## The Pipeline Solution

It is in light of the FDA’s Critical Path Initiative that we can best understand the significance and goals of its *Guidance for Industry, Investigators, and Reviewers: Exploratory IND Studies*, issued on January 12, 2006 [10]. According to the FDA, this guidance has several goals in regard to planning exploratory studies in humans. One is to identify likely pre-clinical and clinical approaches suitable for such exploratory studies. Another is to outline what information pertaining to chemistry, manufacturing and controls needs to be considered for exploratory studies, including those of closely related drugs or therapeutic biological products. A third and pivotal goal is to remind investigators of the considerable flexibility in the amount of data required when submitting a research proposal under current regulations for an investigational new drug (IND) application (21CFR 312). Whether in regard to the goals of the research, the specifics of the human testing being proposed or the anticipated probability of harms to humans consequent to this testing, it is the position of the FDA that investigators have mistakenly been inclined to provide more supporting information in an IND application than the regulations stipulate. And seeing this mistaken tendency as symptomatic of the “pipeline problem” detailed in the Critical Path Initiative, the FDA issued this particular guidance “to clarify what manufacturing controls, pre-clinical testing, and clinical approaches can be considered when planning limited, early exploratory IND studies in humans” [11].

In pursuit of the first goal cited above, the guidance includes examples of early Phase I (now commonly referred to as Phase 0) models that comply with current regulations and combine customary protection of human subjects with fewer of the other customary research resources in a way that promotes a more efficient development of promising drugs and biologics. Accordingly, an exploratory IND study refers to a clinical trial that is conducted early in Phase I, that treats study participants with a very low dose of drug given over a short (7–11 days) period of time, without therapeutic or diagnostic intentions. In contrast with the dose escalation, safety and tolerance goals associated with Phase I studies and the traditional start of a drug-development program, here is a study model, conceptually and methodologically designed solely with the goal of determining the likelihood of the agent’s further development.

## Exploratory IND Studies

Throughout this guidance, The FDA is at pains to differentiate the nature, goals and regulatory requirements for the exploratory IND study from those of the traditional Phase I study. In keeping with the stipulated limitations of the exploratory model, the guidance presents it more specifically as a means to determine if the drug’s mechanism of action as observed in an experimental system can be replicated in humans. Accordingly, a complementary goal is to generate reliable pharmacokinetic (PK) data. Then on the basis of confirmed PK and pharmacodynamic (PD) characteristics, the exploratory model can be instrumental in selecting the most promising of several candidate agents designed to interact with selected therapeutic targets in humans. The earlier the selection, the more successful the study will be judged because it will have involved “dosing a limited number of subjects with a limited range of doses for a limited period of time” [12].

Unlike the traditional IND study, which focuses on the likely outcome of the clinical study, the exploratory IND study is strictly limited to a study without whose findings there cannot be any possible development of the selected agent or agents. As a result, the justification for the study depends on the reasons for selecting and studying a particular agent or combination of agents. And upon completion of the study, it is the expectation of the FDA that the agent or agents will be withdrawn.

In keeping with a study rationale of this nature, the FDA recommends single- and multiple-dose studies as suitable approaches. In the former, it envisages the administration of sub-pharmacological or pharmacological doses to a restricted number of subjects in order to generate PK data or perform imaging studies. In the latter, the goal would be to generate PD data, while stopping short of determining the subject’s tolerance of the agent.

Along with these recommendations, the guidance includes a discussion of agent chemistry, manufacture and controls (CMC). It insists that an exploratory IND application contains information on the risk of harm to subjects from the chemistry or manufacture of the agent based on pre-clinical studies. Where it is believed to exist, the application must identify the steps to be taken to monitor risk. To this end, the guidance calls for a description of the physical, chemical and biological characteristics of the agent, its dosage form, routes of administration, quantitative composition, stability and analytical characterisation.

The guidance closes with a discussion of safety. Once again, it emphasises that in contrast with the traditional IND application, in the case of exploratory INDs, the standards for toxicology assessment are more limited so as to match the more limited reach of an exploratory clinical study. For instance, even if a particular exploratory IND study might entail pharmacological outcomes, its goal would not be to determine the maximum tolerated dose (MTD). And given that, the FDA wishes to underscore a central point of the guidance. Namely, those levels of pre-clinical testing for safety are to be in direct proportion to the reach and identified objectives of the exploratory study itself. Among study objectives that might be used as criteria establishing levels of safety testing the guidance includes confirmation that expected mechanisms of action are demonstrable in humans: the binding characteristics of the experimental agent; PK and metabolic findings; and the effect on a potential therapeutic target compared with other therapies.

## The Ethics of the Phase 0 Construct

It is clear from the way the Phase 0 construct is described in both the *Critical Path Initiative* and the *Guidance* that the FDA is promoting it as a means to achieve an end, namely, solving the “pipeline problem”. If so, then an ethics assessment of the construct should start by asking whether the end justifies the means in moral terms. To answer this, we need to determine what, if any, is the moral standing of the pipeline problem, the solving of which would constitute a moral good. As long as the pipeline problem persists, research will frustrate investigators by taking longer, being more expensive, resulting in more dead ends and ultimately failing to meet the needs of patients. However, solving the pipeline problem will mean that we have reversed each of these and enabled researchers to respond effectively to the needs of patients. If this is the case, it seems reasonable to say that solving the pipeline problem is a good thing to do. Now as long as we are commending solving the pipeline problem for being beneficial, directly or indirectly, for patients, we are commending it for moral reasons [13]. In which case, to say it is good is to say something more than descriptively saying what solving the pipeline problem is like [14]. It is to say something prescriptively. Now “good”, when used in ethics has both a descriptive and a prescriptive function [15]. As a result, on the philosophical principle that descriptive statements are subordinate to prescriptive statements, to say that solving the pipeline problem is a good thing is to say that it ought to be done [16]. And were one to say that solving the pipeline problem is a good thing to do but I will not do it, one would find that odd and expect an explanation [17].

If the goal of solving the pipeline problem has moral standing and should be pursued, does it follow that any means, whether good or bad, selected to achieve this goal gains moral standing by virtue of this means–end relationship? Can we, in other words, stipulate something like the following: since solving the pipeline problem is a good thing, and since Phase 0 studies may help to solve the problem, we ought therefore to conduct Phase 0 studies? At one level we can, since, according to Mortimer Adler [18], the only thing justifying a means is the end it is designed to achieve. But the end we are pursuing could be, as he pointed out, stealing or murder. In that case, he continued, since there is no justifying or making right the pursuit of such an end, it follows that such an end cannot be used to justify any means whatsoever to achieve it. But what, Adler asked, of good ends? Since it is justified morally to pursue good ends, like solving the pipeline problem, is it justified to use any means in their pursuit? Mortimer would, I believe, have agreed. As he noted “…if the end is, etc., “for if the end is really good, and if the means really serves the end and does not defeat it in any way, then there can be nothing wrong with the means. It is justified by the end and we are justified in using it” [19]. He added, however, that were some activity unethical in itself, it could not, as it were, become ethical in virtue of the end it was selected to serve. It would only compromise the integrity of that end.

In light of this analysis, what can be said to justify employing Phase 0 studies as a means to solve the pipeline problem? That will depend on what the moral standing of the Phase 0 construct is. In one important respect, the construct fails to meet the ethical standards set by the Belmont Report (1979) for the conduct of trials involving human subjects. This is the standard of direct benefit to the study participant. Since by definition, a Phase 0 study calls for microdosing over a very short period of time, there is no expectation of achieving any direct therapeutic benefit for the patient. By the same token, there is virtually no risk of harm to participants either. Any expectations for indirect benefit are similarly limited. Since the duration of participation – 7–11 days – is so brief, it is hard to see what psychological benefit, for example, might be experienced by the participant. And if a participant was hoping to enjoy any benefit from the fact that, by participating in a research study, she was taking control of her medical fate, it is hard to see that taking place through something so fleeting as a Phase 0 study. That leaves benefit to others as the only remaining standard stipulated in Belmont for purposes of justifying ethically a clinical study. However, Belmont did not view benefit to others as an independent standard but as one of three considerations of benefit. But in the absence of direct benefit compensating for the probability or risk of harm, neither indirect benefit nor benefit to others justifies a clinical trial. This position was confirmed by the World Medical Association Declaration of Helsinki which stipulated that the interests of individual human participants in clinical studies must always be placed above any of the interests society at large might seek to benefit from those same studies [20]. As the National Bioethics Advisory Commission (NBAC) put it, the concept of “benefit to others” that might result from clinical studies offering no reasonable expectation of direct benefit “poses in the most dramatic form the conflict between the societal interest in the conduct of important and promising research and the interests of potential subjects” [21]. Although intended for Phase I, II, and III studies, these words have even more relevance to Phase 0 studies where the conflict between the two sets of interests is considerably less ambiguous. And while technically, Phase I studies are understood to have no expectations of direct benefit, it is still reasonable, given their history of efficacy, to conduct them with therapeutic intent. Although a Phase 0 is described as a very early Phase I, it must, however, be understood as distinctly different from the Phase I construct. The difference is based on two significant considerations. The first is that the Phase 0 construct cannot be justified in consideration of benefit. The second is that the construct poses virtually no risk of harm to participants. In the absence of benefit and harm, there is no point in considering it, at least therapeutically, as an end in itself. The alternative, which has already been broached, is to consider more thoroughly the construct for its science simply and for the possibility that therein we may find ethical warrant for the construct as a means in the FDA’s plan to solve the pipeline problem.

## Phase 0 As Science

To this end, we would do well to turn to Jacob Bronowski’s discussion of the nature of science [22]. He maintained that science begins with the creation of concepts, such as mass, and from there proceeds to test them for their empirical truth to fact. Paralleling this, Anthony Murgo *et al* [23], in discussing the design of Phase 0 cancer clinical studies, maintain that the first step is to consider what agent would be appropriate. They add that if the goal is to evaluate the effect of the agent on the selected biomarker, then applicable criteria for deciding on the agent include a pharmacodynamic (PD) end point, a credentialed biomarker, a wide therapeutic window, the expectation that biomarker modulation will occur at non-toxic dose levels and limited exposure levels with a relatively small sample of between 10 and 15 patients. Whether considering novel therapeutics, imaging probes and biomodulators, these same criteria would be applicable. Based on his analysis of science in general, Bronowski concluded that “[t]ruth is the drive at the centre of science; it must have the habit of truth, not as a dogma but as a process” [24]. He was saying that the values of science have come from the practice of science as the necessary conditions of scientific practice. He might well have been speaking of the Phase 0 construct as Murgo and colleagues have described it.

But the parallel does not end there. When Bronowski described the habit of truth, not as dogma but as process, he understood truth to be something we find, not something we are given. One consequence of this is the need for what he called a society of scientists recognisable for its independence, originality, dissent and tolerance. “Science confronts the work of one man [sic] with that of another and grafts each on each; it cannot survive without justice and honour and respect between man and man”[25]. These values are so integral to the practice of science, that if they did not exist, the society of scientists would, Bronowski insisted, have had to invent them to make the practice of science possible.

To a particularly relevant degree, the implications of Bronowski’s societal conception of science applies to the Phase 0 construct, in particular, its design. As Murgo and colleagues view it, the design calls for “a rational transition from pre-clinical to clinical development” [26]. This, they believe, includes developing a methodology sufficiently coherent to model several complementary steps including tissue acquisition, handling and processing; validating a biomarker analytic assay; and assessing the effect of the drug on the biomarker, and the pharmacokinetic and pharmacodynamics relationships. “The seamless transition from pre-clinical to clinical development is critical to the design of phase 0 trials and requires close collaboration between laboratory, drug development, and clinical scientists” [27].

If we can presume collaboration, we do so because, as Bronowski observed, the nature of science is such that it values the search over the discovery. “In the society of scientists, each man by exploring for the truth has earned a dignity more profound than his doctrine” [28]. But only on condition, he adds, that the scientist approaches his/her theory and practice with the respect born of self-honesty. And it is from this self-honesty that Bronowski finally draws the scientist’s moral, and indeed Adler’s moral, that there is no distinction between ends and means. To underline this crucial point he cited mathematician WK Clifford: “…if I let myself believe anything on insufficient evidence, there may be no great harm done by this mere belief; it may be true after all… But I cannot help doing this great wrong towards Man, that I make myself credulous. The danger to society is not merely that it should believe wrong things, though that is great enough; but that it should become credulous.” (29).

## Phase 0 and Credulity

Bronowski explains that Clifford’s point hinges on the phrase, “it may be true after all”, pointing out that while some might justify their actions with such credulity, the scientist cannot since the word true cannot have such a meaning in science. “The test of truth is the known factual evidence and no glib expediency . . . can justify the smallest self-deception in that” [30]. In pursuing the several economies expected from the Phase 0 construct, the risk of credulity may be real. Here, Murgo and colleagues may have expressed their own caution against credulity, noting the significant limitations of the construct and advocating its narrower over its broader application. “Not all agents are appropriate for Phase 0 testing” [31]. They also point out that the resources needed for the pre-clinical and clinical components of the construct, those, for example, assessing effects on selected biomarkers, are not widely available. Furthermore, accrual of patients to non-therapeutic studies may prove problematic. And the minimum requirements for the scientific validity of the Phase 0 construct include the following: a laboratory for the development of PD assays; staff with the appropriate training in the development and validation of analytic assays for biomarkers; facilities for PD and PK studies done in real time on human tissue; a system for the collection and processing of biospecimens; and finally an integrated team of laboratory and clinical investigators, expert in conducting early-phase studies. Translated into Bronowski’s language, these cautionary words on requirements can mean that to silence one scruple concerning means, be it an agent, an analytic assay-development laboratory, or a system for procuring biospecimens, is to infect ourselves as scientists and our ends.

The Phase 0 construct promises to be a welcome development in clinical research as long as we see it for what it is scientifically and what it should be ethically. However, different as its scientific constitution is, so also its ethics constitution. Scientifically, it has been developed as a means to a very specific end. And it is on that basis that an ethics critique of the construct must be developed. The concept of benefit, particularly direct benefit, which has been central to the standard ethics critique of the other research phases, is not relevant to Phase 0. But a critique based on the means-end relation as understood by Adler provides a reasonable alternative to understanding what it should be, given that relation. And placed within Bronowski’s formulation of an ethic of science derived from the practice of science, it affords a robust mechanism with which to address the ethical challenges posed by the Phase 0 construct since “the fine structure of science make(s) the grain of conscience” [32].
